# Improving nurses’ mental health through an online Acceptance and Commitment Therapy intervention: an exploratory pilot study across two healthcare contexts

**DOI:** 10.1186/s12912-026-04587-y

**Published:** 2026-05-13

**Authors:** Eveline Frey, Yuen Yu Chong, Wai Tong Chien, Andrew T. Gloster

**Affiliations:** 1 Faculty of Psychology, University of Basel, Basel, Switzerland; 2 Division of Medicine, Chinese University of Hong Kong, The Nethersole School of Nursing, Hong Kong, China; 3 Division of Clinical Psychology, University of Lucerne, Lucerne, Switzerland; 4 Faculty of Psychology, University of Basel, Missionsstrasse 62A, Basel, CH-4055 Switzerland

**Keywords:** Nursing professionals, Acceptance and commitment therapy, Psychological flexibility, Digital psychological interventions, Healthcare contexts, COVID-19 pandemic

## Abstract

**Background:**

The COVID-19 pandemic negatively impacted healthcare workers’ (HCWs) mental health, particularly nurses, due to their demanding frontline roles. Easily accessible psychological interventions are needed to support their well-being, especially during crises like lockdowns. This study aimed to support nurses during the pandemic through online-based Acceptance and Commitment Therapy (ACT) interventions. While ACT, a third-wave cognitive-behavioral approach, has shown effectiveness in reducing work-related stress, its impact on nurses across cultures remains unexplored.

**Methods:**

We used a multi-stage process (Delphi Method) to develop online-based ACT interventions. This process included a needs assessment through semi-structured interviews with nurses (Hong Kong: *n* = 8; Switzerland: *n* = 20) and focus groups conducted to evaluate the interventions before implementation. The intervention — consisting of two half-day workshops — was then tested in a pilot study with nurses from Hong Kong (*n* = 7) and Switzerland (*n* = 10).

**Results:**

Levels of stress, emotional exhaustion, anxiety, and depression decreased, while levels of psychological flexibility increased over time. No regional interaction effects were observed, except for depression, which declined more significantly among Hong Kong nurses than Swiss nurses.

**Conclusions:**

This was the first study to examine an online ACT intervention for nurses across different cultural backgrounds. Findings suggest ACT effectively improves nurses’ mental health, but further research is needed to confirm its effectiveness in varied settings due to the study’s exploratory design and limited sample size.

**Trial registration:**

The study was prospectively registered at ClinicalTrials.gov on 10.03.2021 (ID: NCT04821037), prior to data collection. In addition, the study was retrospectively registered with the Open Science Framework (OSF; registration number tpgd6) on 12.06.2025 to provide additional documentation and materials.

**Supplementary Information:**

The online version contains supplementary material available at 10.1186/s12912-026-04587-y.

## Background

### Mental health of nurses during the pandemic

The negative effects of the COVID-19 pandemic on the mental health of the general population have been documented in various studies [1–4]. However, Healthcare workers (HCWs) were identified as a group at particular risk of developing physical/mental problems as a result of working directly or indirectly with COVID-19 patients [5]. Challenging working conditions, such as exposure to the threat of transmission [6], inadequate protective equipment, long working hours, understaffing [7–9] and severe stress, high emotional load, concerns of being infected or infecting their relatives, insufficient workplace support, and the absence of effective supportive treatments [10, 11] have been associated with an increased risk of developing mental health symptoms among HCWs. Results from a meta-analysis showed that 62.5% of HCWs exposed to COVID-19 reported general health concerns, 43.7% fear, 41.2% fatigue, 34.6% headaches, 37.9% insomnia, 37.8% psychological distress, 34.4% burnout, 29% anxiety, 26.3% depressive symptoms, 20.7% post-traumatic stress disorder features, 16.1% somatization and 14% stigmatization feelings [12]. In addition to the increase in psychological symptoms, the pandemic also had a negative impact on the well-being of HCWs. Findings from a study with HCWs and 556 participants revealed that 40% of the participants reported a WHO Well-Being index score below 13, indicating poor mental well-being and the need for further assessment for depression [13]. Frontline workers, such as nurses, are particularly vulnerable to negative consequences and psychological symptoms during the pandemic, as they are most frequently exposed to stress factors. Indeed, nurses were more heavily impacted by the pandemic than other healthcare professionals, as reflected in the higher prevalence of mental health issues [14–16]. However, this burden is likely not uniform across all settings. Structural and organizational factors – such as resource availability, staffing levels, and prevailing cultural norms – shape psychological distress and well-being and may account for the variations across different healthcare contexts.

Furthermore, certain systemic barriers make it difficult for nurses to seek support. These include the persistent stigma regarding mental health within the profession [17, 18], limited access to psychological services, and a work environment that often discourages help-seeking behavior [19–21]. Importantly, elevated psychological strain among healthcare workers has also been reported beyond the COVID-19 pandemic. For example, previous large-scale health crises, such as the severe acute respiratory syndrome (SARS) outbreak (2002–2004) [22] and the West African Ebola outbreak (2013–2016) [23], have similarly been associated with increased psychological strain among healthcare workers [24, 25]. Taken together, these events highlight the need for strategies to mitigate the adverse effects of mental health symptoms among HCW. One strategy are therapeutic interventions, delivered by trained mental health professionals. These interventions must be easily and early accessible, tailored to the specific needs of healthcare workers, applicable across different crisis contexts, and feasible under conditions such as lockdowns. Although limited research exists on the effectiveness of psychological interventions for improving healthcare workers’ mental health during crises such as the COVID-19 pandemic, findings from a systematic review suggest that early psychological interventions may be effective for frontline staff [26]. Continued implementation and evaluation by service providers are needed to establish evidence-based approaches for future crises.

### Acceptance and commitment therapy

One possible approach for such interventions is Acceptance and Commitment Therapy (ACT). ACT is a psychotherapeutic approach rooted in behavioral therapy which uses six skills to promote psychological flexibility. Psychological flexibility is described as a process of noticing experiences in the present moment without judgement, and maintain or change behaviors that serve the pursuit of value-driven goals [27]. Psychological flexibility is a well-established predictor of long-term psychological health [28, 29] and has been identified as a central underlying mechanism for improvements in mental health in ACT interventions [30–32]. The six core skills for developing and enhancing psychological flexibility are described by Hayes [30] as follows: (a) acceptance, (b) cognitive defusion, (c) self as context, (d) being present, (e) values and (f) committed actions. The skill of acceptance means being open to experiencing thoughts, feelings, memories or situations [27], even when they are painful. It offers an alternative approach of experimental avoidance, which means avoid or suppress painful thought or emotions. Being present and practicing defusion means being aware of one’s thoughts, feelings, or sensations as they arise in the present moment, without allowing these to dictate one’s actions by their internal content. The skill of self-as-context is the ability to consciously perceive one`s experience from the past and the present without attachment to them. Instead, it involves adopting an observing perspective towards these experiences, which is why the skill is often also termed the “observing self”. Finally, the skills values and committed actions are about becoming aware of one’s core values, what truly matters in life- and aligning one`s actions and behaviors accordingly (value-driven-actions). An ACT intervention focuses on teaching strategies and techniques to cultivate these six core skills.

Although ACT interventions do not primarily aim for symptom reduction—focusing instead on acceptance and mindfulness strategies to support values-based action despite difficult emotions [27] — it is hypothesized that nurses who adopt these strategies may experience lower levels of burnout, depression, and anxiety over the long term. Given that nurses are repeatedly exposed to uncontrollable stressors, acceptance-based strategies may prove more sustainable than a traditional focus on symptom suppression. Furthermore, value-based interventions could help nurses reconnect with the professional values and motivations that initially drew them to the field. This alignment may foster a greater sense of purpose, helping to sustain them even under conditions of high workload and chronic stress.

Several systematic reviews have shown the positive effect of ACT on clinical and non-clinical samples [33, 34]. ACT interventions have also proven to be effective in the context of employee stress-reduction [35], for example in public health sector workers [36], local government employees [37], social workers [38], and school teachers [39]. ACT, in the form of self-help interventions, was also found to be effective, leading to a reduction in burnout and stress among individuals from various professional backgrounds [40]. Currently, there is a limited number of studies on the effectiveness of ACT interventions for healthcare workers, particularly for nurses. Since nurses are considered a particularly vulnerable occupational group within healthcare workers, further research is needed to demonstrate the effectiveness of ACT interventions specifically for this group.

Although limited in number, the existing studies demonstrate promising results regarding the effectiveness of ACT interventions among nursing professionals. A cross-sectional study conducted with 142 nurses from England showed that all six core skills of ACT were positively correlated with compassion satisfaction a negatively correlated with stress, burnout and compassion fatigue. Among these, the core skills of acceptance, mindfulness, and value-based processes were found to be the most effective [41]. A study conducted in 2016 with nursing students also found that ACT interventions led to a reduction in burnout, stress, and experiential avoidance [42]. Two studies involving nursing professionals from acute care settings showed that ACT interventions contributed to a reduction in stress and burnout [43], as well as improvements in psychological flexibility and well-being [44]. There are also two studies demonstrating the effectiveness of ACT interventions for nursing professionals working in long-term care settings. Zarvijani et al. [45] found that ACT interventions for psychiatric nurses led to a reduction in burnout and stress, as well as to an increase in psychological flexibility. Similarly, the results from Montaner et al. [46] showed that nurses working in dementia care units experienced a reduction in emotional exhaustion and an improvement in psychological flexibility through ACT interventions.

In addition to the limited number of studies that specifically examine the effectiveness of ACT interventions among nursing professionals, there is currently a lack of research investigating whether context-specific factors — such as region, healthcare systems and working conditions, or cultural differences — influence their effectiveness. Consequently, it remains unclear whether ACT interventions are universally effective for nurses across different regions and cultures, or whether these contextual factors significantly impact their efficacy. Addressing this question is essential in order to tailor health programs and interventions to specific contextual conditions.

Based on these considerations, this study aimed to achieve two primary objectives: first, to contribute to the growing body of evidence regarding the effectiveness of ACT interventions for nursing professionals; and second, to determine whether such interventions remain effective across diverse healthcare contexts. To this end, we conducted a pilot study involving nurses from Hong Kong and Switzerland—two regions specifically chosen to represent a broad spectrum of cultural and organizational contexts. The selection of these locations allows for a robust comparison between more collectivist and more individualist cultural orientations, as well as distinct healthcare system structures and varying cultural attitudes toward mental health and stigma. This cross-contextual approach is essential for evaluating the broader applicability and feasibility of the ACT framework in global nursing care. With respect to our first aim, we hypothesized that the ACT interventions would lead to reductions in stress, burnout, depression, and anxiety (primary outcomes), as well as improvements in mental well-being and psychological flexibility (secondary outcomes and ACT-specific constructs). Regarding the second objective, we explored whether ACT interventions would be associated with beneficial outcomes for nursing professionals across different healthcare contexts, including Hong Kong and Switzerland.

## Methods

### Study design

We developed and evaluated an ACT intervention in two phases. Phase 1 consisted of the intervention development, during which we used a multi-stage survey process based on the Delphi method [47]. The Delphi method is a systematic, multi-stage approach used to gather feedback from experts, in this case nursing professionals. Applying a Delphi-based approach supports the development of interventions that are closely aligned with the needs of the target group and enhances their usability and acceptability [48, 49]. Phase 2 consisted of a pilot study with three measurement points.

### Phase 1: Qualitative intervention development

A modified Delphi approach was used to guide the user-centered development of the ACT-based intervention. The process was exploratory in nature and aimed to iteratively integrate feedback from the target group rather than to achieve formal consensus using predefined quantitative thresholds.

The modified Delphi process consisted of several iterative stages. First, semi-structured interviews were conducted to identify key stressors, mental health challenges, and perceived support needs among nursing professionals. Based on these findings and prior research on employee-focused interventions, preliminary ACT-based intervention components were developed.

In a second stage, the proposed intervention components were presented and discussed in online focus groups. Participants were invited to provide feedback on the relevance, feasibility, and perceived usefulness of the proposed components. Following the focus groups, participants completed an evaluation questionnaire that included quantitative ratings as well as optional open-ended questions for additional comments.

Participants in the modified Delphi process were nursing professionals working in hospital settings in Hong Kong (*n* = 8) and Switzerland (*n* = 20). They were recruited via flyers, email invitations, and professional networks, primarily in large public hospitals. Participation was voluntary and took place in the participants’ free time. Eligibility criteria included being at least 18 years old, holding a nursing degree, being employed at a hospital in Hong Kong or Switzerland at the time of the study, having access to an electronic device, and understanding Chinese or German. No external expert panel was included, as the primary aim was to develop an intervention grounded in the perspectives and needs of the target group.

### Interviews

After agreeing to participate, a date for the interview was arranged with each participant. Study information was sent via email, and written informed consent was obtained prior to participation. Interviews were conducted via Zoom over a three-week period in December 2020 and lasted approximately one hour.

The interviews were conducted by three members of the study team who had received prior training from the study manager. A semi-structured interview guide was used, informed by the Acceptance and Commitment Therapy (ACT) mode. The interview consisted of two parts. In the first part, participants responded to eight open-ended questions addressing the following topics: motivation for choosing the nursing profession, perceived work-related stressors and coping strategies, perceived support, desired changes in the work context, and values and commitment related to their professional role. To further explore values in a structured manner, personal values were assessed. Participants were asked to rate the importance of ten life domains on a scale from 1 (not important at all) to 10 (extremely important). The second part of the interview was designed to systematically identify difficult emotional experiences, stress reactions, and patterns of psychological flexibility and inflexibility. Participants were asked to reflect on the emotional versus organizational nature of their work-related challenges and to estimate their relative impact. They were further asked to rate the extent to which specific emotional stressors (e.g., dealing with suffering, ethical dilemmas, fear of infection, unpredictability) and stress reactions (e.g., sleep problems, anxiety, somatic symptoms, social withdrawal) applied to them. Additional open-ended questions explored perceived changes in stress experience since the COVID-19 pandemic and individual stress responses. To identify intervention needs, participants were also asked to rate the perceived helpfulness of various support options and ACT-consistent intervention elements (e.g., mindfulness exercises, learning to relate differently to difficult thoughts and feelings, values-based action). The interview concluded with open-ended questions allowing participants to voice additional concerns or messages they wished to share.

The full interview protocol is provided in Additional file 1. All interviews were audio-recorded and transcribed verbatim.

### Qualitative analysis and consensus procedure

Interview data were analysed using a directed qualitative content analysis guided by a theory-informed framework [47] based on the six core processes of the ACT Hexaflex. This approach was chosen to systematically identify indicators of psychological inflexibility, difficult emotional experiences, and values relevant to intervention development. An analytic matrix was developed in Microsoft Excel, with interview questions represented along the horizontal axis and predefined coding categories (ACT processes and difficult emotional experiences) along the vertical axis.Transcripts were reviewed line by line, and relevant text segments were extracted verbatim and assigned to the corresponding cells of the matrix. Coding focused on explicit and implicit references to experiential avoidance, cognitive fusion, values, committed action, and related emotional experiences. Following this deductive coding step, data from all interviews were pooled and examined for recurring patterns across participants. Frequently reported emotions, stress reactions, and coping patterns were grouped into higher-order clusters (e.g., recurrent feelings of hopelessness or difficulties disengaging from work-related thoughts).These clusters were used to identify central support needs and to inform the selection and tailoring of ACT-based intervention components.

Coding and analysis were conducted independently by four researchers (ATG, EF, FC, CP) with backgrounds in clinical psychology, psychology training, and nursing. To enhance analytic rigor and reduce individual bias, regular consensus meetings were held to compare coding decisions, discuss discrepancies, and refine the analytic framework until agreement was reached.

### Focus groups

The findings from the interviews and the proposed intervention components were presented to the same participants who had taken part in the earlier interviews during focus group sessions conducted in both Hong Kong and Switzerland. After the focus groups, participants rated the proposed interventions in terms of perceived helpfulness and evaluated preferred intervention formats (e.g., weekdays versus weekends, single session versus multiple workshops). The evaluation questionnaire is provided in Additional file 2.

Participants who completed the focus groups and evaluation questionnaire received compensation of 30 CHF and a thank-you card.

Based on convergence of feedback across interviews, focus groups, and evaluation questionnaires, and through discussion within the interdisciplinary study team, the intervention components for the pilot study were selected. Formal percentage-based agreement thresholds were not applied, consistent with the exploratory nature of the intervention development.

Findings from the modified Delphi process directly informed the final intervention design. For example, difficulties with mentally disengaging from work-related thoughts were frequently reported. For instance, one participant remarked, *“I can’t switch off after work “* while another participant stated,*“I want to learn strategies to detach from stressful work situations while I am at home.”* In response, cognitive defusion exercises were included to support more flexible ways of relating to distressing thoughts. Similar principles guided the selection of other ACT-based exercises. In addition, participants’ feedback informed decisions regarding the overall structure and delivery format of the intervention, including the division into two workshops, session length, and scheduling preferences (e.g., weekdays versus weekends).

The final ACT intervention included both psychoeducation and ACT techniques and consisted of several modules covering the core processes of ACT (Table 1). The complete workshop structure is provided in Additional File 3.

**Table 1:** Content of ACT interventions

Module	Key components	Purpose
Day 1: Introduction	Introduction of trainer and participants; structure and goals of the ACT intervention study; short introduction on ACT	Orientation, establishment of group safety, introduction to ACT model
Acceptance, self as context	Experience-based exercise for acceptance and self as context; group discussion	Enhancing openness to internal experiences and perspective-taking
Being present, self as context	Experience-based exercise: being present and self as context; group discussion	Strengthening present-moment awareness and psychological distance from thoughts
Values and committed action	Exercise on values; psychoeducation on values, group discussion, exercise for values; group discussion, exercise for values and committed actions, group discussion, tainer input	Clarifying personal values and linking values to meaningful action
Closing day one	Conclusion of day one; answering open questions; homework: exercise on values and committed actions	Consolidation of learning and transfer to daily life
Day 2 : Introduction	Warm-up; short repetition of day one	Reactivation of prior content and experiential engagement
Values and committed action	Reflection homework: what experiences were made by doing the ACT exercise?	Strengthening values-based reflection and learning from experience
Acceptance, cognitive defusion	Psychoeducation for acceptance and defusion; group discussion, experience-based exercise for acceptance and defusion	Reducing experiential avoidance and cognitive fusion
Values and committed action at work and in personal life	Group task: group discussion: what are advantages if we knew our team values and try to act accordingly?; individual task: create a 4-week plan with value-based goals and actions for the next 4 weeks	Promoting sustained values-based action in work and personal contexts
End	Repetition of the workshop: summary of all interventions, asking for feedback to the workshops and answering open questions	Integration of ACT processes and intervention closure

Note: Summary of the two-day ACT intervention including all experiential exercises and group discussions

### Phase 2: Pilot study

In the second phase, the intervention was evaluated for applicability, feasibility, and potential effectiveness through a pilot study consisting of two 3.5-hour online ACT workshops conducted in groups, with three measurement time points. The study design is presented in Fig. 2.

**Fig. 1:**
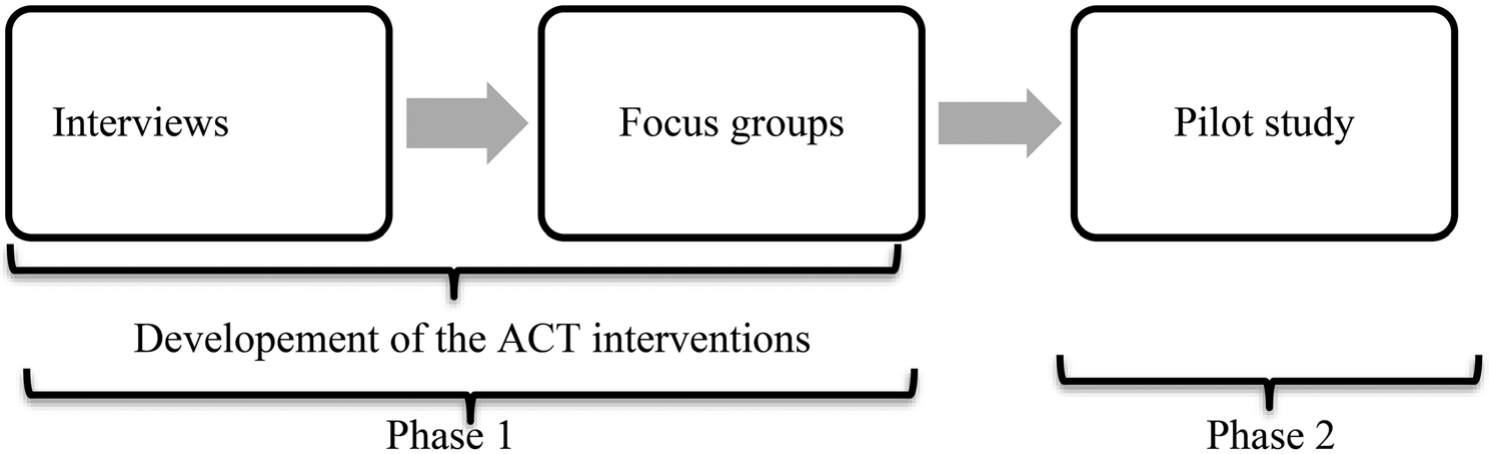
Study design

 The ACT intervention study was conducted as a pilot study in July 2021 during the fourth wave of the COVID-19 pandemic, with nursing professionals from Hong Kong (*n* = 7) and Switzerland (*n* = 10). The ACT intervention study was conducted through two 3.5 h online workshops, which took place one week apart. For recruitment, hospital management and supervisory staff from different hospitals in Switzerland and Hong Kong were contacted and informed via an information letter about the upcoming ACT group training for nurses. In both regions, one supervisor from a large public hospital agreed to participate in the program with their team, provided that team members met the inclusion criteria for the pilot study (being at least 18 years old, holding a nursing degree, working at least 40% in a somatic nursing team, speaking German or Chinese, and having access to an electronic device). Individual team members were not contacted directly prior to this agreement. Only after the supervisor had agreed to participate and identified eligible team members were these individuals contacted by the study manager, received detailed study information, and provided informed consent. All participants worked within the same team and were able to attend the workshop during working hours with approval from their supervisor.

The workshops were delivered via the online platform Zoom and were delivered by qualified facilitators in both study sites. In Hong Kong, the workshops were conducted by a professor of psychology and a PhD student in psychology. In Switzerland, the workshops were delivered by a PhD student with clinical experience and qualification as a registered nurse, together with a master’s student in psychology. All facilitators received prior training from a professor of clinical psychology with specialization in Acceptance and Commitment Therapy (ACT) to ensure consistent delivery of the intervention across sites. The intervention was delivered using the same structure, content, and sequence across both study sites. Workshops were conducted by native-speaking facilitators in each country to ensure appropriate language use and contextual understanding. No site-specific changes were made to the core ACT components. Before each workshop, participants received an access link and workshop materials via email.

The first workshop day started with a mindfulness-based exercise to introduce an experiential approach from the outset. The main aim of the workshop was to convey ACT processes primarily through experiential exercises, based on the assumption that these foster more involvement than a strong focus on cognitive load, psychoeducation, or purely theoretical content. This was followed by a brief psychoeducational section explaining why avoidance, although often experienced as a coping strategy, is unhelpful in the long term, and why ACT focuses on alternative ways of living a meaningful life despite difficult emotions. The ACT processes covered on the first day were mindfulness/being present, values, and values-based action. On the second workshop day, acceptance and defusion were addressed, and values were revisited and further deepened. Between the workshops, participants were encouraged to practice the ACT exercises at home and share their experiences during the next session. Mental health outcomes were accessed pre (one week before the first workshop, T0), post (in the week after the second workshop, T1) and one month follow-up (T2). The same mental health outcomes were assessed for all three time points (T0, T1, T2), in the first questionnaire, the demographic data were also collected. Participants who completed the two workshops and all three questionnaires received a financial compensation of 50 CHF.

### Measures

Primary outcomes and ACT process measures were administered online and self-completed by participants at baseline, post-intervention, and follow-up.

### Primary outcomes

#### Stress

Perceived Stress Scale (PSS-10). Stress was assessed using the Perceived Stress Scale (PSS-10; (Cohen et al., 1994)). The PSS measures an individual’s appraisal of how stressful situations in their life are. Items ask about people’s feelings and thoughts during the last month. A total score is produced, with higher scores indicating greater overall distress. The Cronbach`s alpha demonstrated good reliability of 0.84 [48].

#### Burnout

Maslach Burnout Inventory (MBI-HSS-22, 7-point Likert scale) was used to measure three dimensions of burnout (Kalliath et al., 2000). The inventory consists of 22 Likert scale questions with subscales of emotional exhaustion (EE), depersonalization (DP), and personal accomplishment (PA). The participants indicated their level of agreement with statements on a 7-point scale ranging from “never” to “daily”. An example item is “I feel drained at the end of a workday”. For each subscale, sum scores are calculated. High scores in EE and DP, as well as a lower score in PA, indicate high degrees of burnout [49].

#### Depression

Patient Health Questionnaire (PHQ-9) [50]. To assess levels of depression the PHQ-9 Scale was used. The PHQ measures the level of depression by asking how often during the last two weeks an individual has felt impaired by certain impairments. A total score is calculated, higher scores indicating higher levels of depression.

#### Anxiety

Generalized Anxiety Disorder 7-item (GAD-7). The GAD-7 assesses the level of anxiety by asking how often an individual has felt impaired by certain symptoms related to anxiety. A sum score is calculated, higher scores indicating higher levels of anxiety. Sum scores > 5 referred to a light or mild level of anxiety, sum score > 10 to a moderate level of anxiety and > 15 to a severe level of anxiety.

### Secondary outcome and ACT processes

Additional measures related to well-being and psychological flexibility, considered an active process within ACT, were included.

### Mental well-being

Mental Health Continuum Short Form (MHC-SF) Short Form (MHC-SF) (Lamers, Westerhof, Bohlmeijer, ten Klooster, & Keyes, 2011). Three commonly referenced dimensions of well-being were measured using the MHC-SF: Emotional Well-Being (EW); Social Well-Being (SW); and Psychological Well-Being (PW). Scores were reversed so that higher scores on each subscale represent lower levels of well-being.

### Psychological flexibility

The PsyFlex questionnaire was used to measure the six processes of psychological flexibility (defusion, acceptance, present-moment-awareness, self-as-context, values, and committed action) (Gloster et al., 2021). The questionnaire consists of 6 Likert scale questions. The participants indicated their level of agreement with statements on a 5-point scale ranging from “very often” to “very seldom”. An example item is “even if I am somewhere else with my thoughts, I can focus on what is going on in important moments”. A reversed sum score was used, and a higher score would indicate greater psychological flexibility.

### Statistical analysis

To account for potential age-related differences between the two country samples, participants were matched on age across sites by excluding individuals outside the overlapping age range.

Statistical analyses were calculated using the IBM SPSS Statistics program (Version 29.0) and R (Version 4.4.3, R Core Team, 2025). Descriptive statistics, reliability analyses, and repeated-measures ANOVAs were performed in SPSS, while additional visualizations were generated using R. A significance level of α = 0.05 (5%) was chosen for all analyses. Given the pilot nature of the study and the small sample size, all quantitative analyses were conducted for exploratory and descriptive purposes. Inferential statistics were used to describe potential patterns, but results should be interpreted with caution and not as confirmatory evidence. No adjustments for multiple comparisons were applied, and the analyses were not intended to formally test hypotheses. A mixed design ANOVA was conducted to evaluate the effects of time point (T1, T2 and T3) and region (Switzerland and Hong Kong) on burnout, stress, depression, anxiety, mental well-being and psychological flexibility. Effect sizes (partial η²) are reported to aid interpretation of observed patterns given the small sample size and high variability in some measures. Significant main effects were followed up using Bonferroni-adjusted or Fisher’s LSD post hoc tests, as appropriate. Given the small sample size and the exploratory character of the study, Fisher’s LSD was used in selected cases. As this procedure is less conservative and associated with an increased risk of Type I error, the findings should be interpreted with caution. The assumptions of normal distribution, homogeneity of covariances and sphericity was examined using the Shapiro-Wilk test, Q-Q plots, Box`s M-test, and Mauchly`s test. The Huynh-Feldt correction was applied when assumption of sphericity was violated. Given the small sample size, assumption checks were interpreted descriptively.

## Results

### Participant enrolment and sample characteristics

Seventeen individuals initially participated i in the study, completing two 3.5 h online ACT training sessions and three online assessments. The final sample included in the analyses consisted of 14 participants (*n* = 7 per region), after participants from both regions were matched by age to enhance comparability between groups.

Participant demographics and baseline characteristics are summarized in Table 2. Age distributions were comparable between groups; given the small sample size, this comparison is reported descriptively.

**Table 2:** Baseline sociodemographic characteristics of participants

Baseline characteristics	Switzerland		Hong Kong		Full sample		χ2 (df)/ t	*p*
	*n*	%	*n*	%	*n*	%		
Age (years) (M, SD)	34.29 (6.85)		29.86 (5.95)		32.07 (6.60)		-1.29	0.22
Gender								
Female	6	85.7	5	71.4	11	68.6	0.42 (1)	0.52
Male	1	14.3	2	28.6	3	21.4		
Marital status							0.53 (2)	0.77
Single	2	28.6	1	14.3	3	21.4		
In a relationship	3	42.9	3	42.9	6	42.9		
Married	2	28.6	3	42.9	5	35.7		
Children ^a^	2	28.6	1	14.3	3	21.4	0.42 (1)	0.52
Years of working experience in nursing								
< 1 year	0	0	1	14.3	1	7.1	7.90 (4)	0.10
1–3 years	0	0	5	71.4	2	14.3		
3–5 years	0	0	0	0	1	7.1		
5–10 years	1	14.3	1	14.3	3	21.4		
> 10 years	6	85.7	0	0	7	50		
Involved in treatment of COVID-19 patients ^a^	7	100	0	0	7	50	14.00 (1)	< 0.001
Feeling overly stressed and / or burnt out at work ^a^	3	42.9	4	57.1	7	50	0.29 (1)	0.60
Went absent from work or was sick due to stress / fatigue ^a^	0	0	2	28.6	2	14.3	2.33 (1)	0.13
Having thought about quitting the job during the COVID-19 pandemic								
Never	2	28.6	5	71.4	7	50	4.29 (2)	0.12
Seldom	3	42.9	0	0	3	21.4		
Sometimes	2	28.6	2	28.6	4	28.6		
Feeling sufficient informed by hospital management during the COVID-19 pandemic								
No, at all	0	0	1	14.3	1	7.1	2.20 (4)	0.70
No, insufficient	1	14.3	1	14.3	2	14.3		
Yes, but not enough	2	28.6	3	42.9	5	35.7		
Yes, sufficient	3	42.9	1	14.3	4	28.6		
Yes, fully	1	14.3	1	14.3	2	14.3		

Note. *N* = 14 (*n* = 7 for each Region). a Reflects the number and percentage of participants answering “yes” to this question. COVID-19, coronavirus, M = mean; N, number of total sample; n, number of sample per group; SD, standard deviation; χ2, Chi-square; t, t- statistics. Due to very small cell counts in several categories, χ² statistics are reported for descriptive purposes only. Percentages should be interpreted in conjunction with absolute frequencies

### Attrition

Dropout was defined as (a) failure to attend both online ACT trainings sessions or (b) failure to complete the questionnaires at all three measurement points. No participants dropped out during the study.

### Intervention effectiveness (Hypothesis 1)

**Fig. 2:**
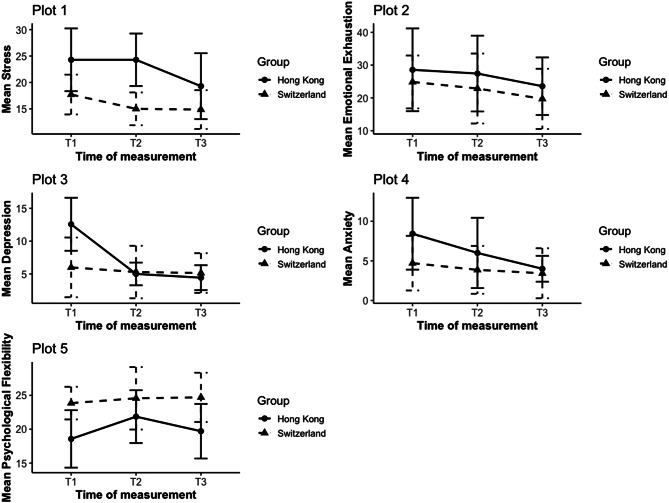
Regional and temporal changes in stress, exhaustion, depression, anxiety, and psychological flexibility. Note: Total *N* = 14 (n = 7 Hong Kong, n = 7 Switzerland). Time of measurement: T1 = prior to the intervention, T2 = one week after the intervention, T3 = one month after the intervention

To explore changes following the ACT intervention the means of all participants were compared across three measurement points (pre-intervention, post-intervention, and one-month follow-up). The mean scores for the primary, secondary, and ACT-specific outcomes are presented in Table 3. Results from repeated measures analysis are illustrated in Fig. 2. For outcomes that did not show notable changes over time, corresponding figures are provided in Additional file 4.

**Table 3:** Means and SDs for mental health variables among Swiss and Hong Kong nurses over time

	*N*	Pretest		Posttest		1-Month FU	
		M	SD	M	SD	M	SD
***Burnout (MBI-EE)***							
Switzerland	7	24.86	8.07	22.86	10.67	19.71	9.18
Hong Kong	7	28.57	12.62	27.43	11.56	23.57	8.78
***Burnout (MBI-DP)***							
Switzerland	7	11.00	5.54	11.57	4.23	11.00	2.65
Hong Kong	7	11.29	3.82	11.29	5.65	8.29	5.30
***Burnout (MBI-PA)***							
Switzerland	7	37.57	4.69	36.71	5.28	35.57	5.70
Hong Kong	7	27.29	7.87	28.71	8.28	28.89	6.82
***Stress (PSS)***							
Switzerland	7	17.71	3.77	15.00	3.11	14.86	3.67
Hong Kong	7	24.29	5.94	20.00	4.97	19.29	6.24
***Depression (PHQ-9)***							
Switzerland	7	6.00	4.55	5.29	4.00	5.14	3.02
Hong Kong	7	12.57	4.04	5.00	1.73	4.43	1.90
***Anxiety (GAD-7)***							
Switzerland	7	4.71	3.45	3.86	3.02	3.43	3.16
Hong Kong	7	8.43	4.54	6.00	4.44	4.00	1.63
***Mental Health Continuum (MHC-SF)***							
Switzerland	7	48.57	8.81	47.43	11.70	49.00	8.08
Hong Kong	7	34.43	5.00	39.57	6.05	37.30	11.04
***Psychologial Flexibility (PF)***							
Switzerland	7	23.86	2.41	24.57	4.61	24.71	3.64
Hong Kong	7	18.57	4.24	21.86	3.90	19.71	4.03

Note. MBI = Maslach Burnout Inventory; MBI-EE = Emotional Exhaustion, MBI-DP = Depersonalization, MBI-PA = Personal Accomplishment (subscales)

### Cross-context comparison of the ACT intervention (Hypothesis 2)

To explore whether changes over time differed between regions, interaction effect between time and region were exmined. For most outcome variables (burnout, stress, anxiety, mental well-being, and psychological flexibility), changes over time appeared comparable between the two regions. For depression, a time-by-region interaction was observed, indicating that patterns of change over time differed descriptively between the Swiss and Hong Kong samples. Given the small sample size, observed regional differences should be interpreted cautiously and primarily as descriptive patterns.

### Primary outcomes

#### Stress

Stress levels showed a decrease over time, with higher mean values observed at baseline compared to post-intervention and follow-up. Post-intervention and follow-up stress levels appeared similar, suggesting that reductions observed after the intervention were largely maintained at follow-up.

#### Burnout

##### Emotional exhaustion

 Emotional exhaustion scores showed a downward trend over time. Lower levels were observed at follow-up compared to baseline, whereas changes between baseline and post-intervention were less pronounced.

##### Depersonalization

 Depersonalization scores remained relatively stable across measurement points, with no notable changes over time.

##### Personal accomplishment

 Levels of personal accomplishment showed no marked changes across the three measurement points.

#### Depression

Depression scores decreased from baseline to post-intervention and remained lower at follow-up. Changes appeared to occur primarily between baseline and post-intervention, with little additional change observed between post-intervention and follow-up.

#### Anxiety

Anxiety levels were higher at baseline compared to post-intervention and follow-up. Reductions observed after the intervention appeared to be sustained over time.

### Secondary outcome and ACT-specific outcome

#### Mental well-being

Mental well-being scores remained relatively stable across all measurement points, with no pronounced changes over time.

#### Psychological flexibility

Psychological flexibility showed an increase from baseline to post-intervention. Scores at follow-up were comparable to post-intervention levels, indicating that initial gains were largely maintained.

## Discussion

The primary aims of this study were to develop ACT-based interventions for nursing professionals and to evaluate their effectiveness on mental health outcomes during the fourth wave of the COVID-19 pandemic. Specifically, stress, burnout, depression, and anxiety were assessed as primary outcomes, while mental well-being and psychological flexibility were examined as secondary outcomes and ACT-specific process variables. Second, we aimed to examine whether ACT interventions demonstrate similar patterns of effectiveness across different healthcare contexts; to this end, we tested our developed interventions among nursing professionals in Hong Kong and Switzerland.

Regarding our first research aim, the results suggest that online-delivered ACT interventions may be associated with improvements in the primary outcomes, particularly stress, emotional exhaustion, depression, anxiety, as well as in psychological flexibility. These results are in line with similar studies. For example, the study by Otared et al. [51] demonstrated that online-based ACT training among HCWs during the pandemic also led to a reduction in anxiety and depression. However, the study by Oatred et al. [51] was conducted with HCWs from various professions, whereas our study included only nurses. Therefore, our findings add preliminary, profession-specif evidence suggesting that ACT may be relevant for nursing professionals, who are particularly at risk for mental health issues among HCWs [15]. Regarding burnout, we observed an improvement in emotional exhaustion, but not in depersonalization and personal accomplishment. These results differ from previous studies that demonstrated improvements across all dimensions through an intervention aimed at reducing burnout [40, 52, 53]. These previous studies were conducted with participants from the general working population or with healthcare personnel comprising multiple professional groups (e.g., physicians, nurses). This was one of the most significant differences between the studies. It is possible that, in the nursing profession, depersonalization and personal accomplishment are more closely linked to the workplace system than emotional exhaustion, especially when compared to other professional groups. For example, depersonalization is sometimes used as a coping strategy that develops in response to lack of autonomy, lack of recognition and prolonged staff shortages and may be more difficult to change. In contrast, emotional exhaustion is often a direct reaction to acute stressors, such as long shifts and high workload demands, and can be alleviated more quickly through rest periods or targeted interventions. In line with our findings, the systematic review by Lee and Cha [54], which examined the effects of intervention to reduce burnout among nurses, found no improvement in personal accomplishment and also suggested that achieving personal accomplishment is strongly influenced by workplace factors, such as control, community or fairness [55], and is therefore more difficult to change through interventions. The assumption that changes in the burnout dimension emotional exhaustion are more easily achievable through interventions is supported by the findings of Hofer et al. [40]. Their study demonstrated that while ACT interventions led to changes across all three burnout dimensions, the clinically significant improvement was substantially greater for EE (53%) compared to depersonalization (21%) and personal accomplishment (15%). In addition to these intervention- and workplace-related explanations, sample characteristics may also be relevant.The Hong Kong nurses in this study were predominantly at an early career stage (1–3 years of professional experience). Depersonalization has been described as a component of burnout that often develops over a longer period of sustained occupational stress [56]. Thus, the absence of observable change in depersonalization may be attributable to the relatively short professional exposure of the sample, rather than to the intervention type per se.

Our study further demonstrated a reduction in stress and an improvement in psychological flexibility. These results align with previous studies showing that psychological flexibility improved through ACT interventions [57] and that perceived stress decreased [42, 45]. Our study differs from previous research as it was conducted exclusively with trained frontline nurses, whereas earlier studies included psychiatric nurses or nursing students. This demonstrates that ACT interventions are potentially helpful in reducing stress even in a demanding sample — namely, practicing nursing professionals, not just those still in training. Furthermore, it shows that ACT is beneficial for frontline healthcare workers, who often experience particularly high stress levels, and not only for those working in long-term care settings. This distinction allows for the development of targeted interventions specifically for frontline nurses. Moreover, our results are consistent with previous findings suggesting beneficial effects of ACT-based interventions in reducing stress and enhancing psychological flexibility in nursing professionals.

Regarding our second research aim — to evaluate the applicability of the ACT interventions across healthcare contexts — the results suggest that similar descriptive patterns of change were observed for nurses in both study sites. These results are consistent with previous findings that have demonstrated the effectiveness of ACT interventions across various cultures and contexts [58]. This suggests that ACT-based approaches may be applicable across different settings, and can be used in multiple cultures and settings. However, to the best of our knowledge, our study is the first to explore and descriptively compare outcome patterns of ACT interventions for nurses from both Eastern and Western healthcare contexts. Therefore, further research is needed to examine potential differences and similarities of ACT interventions among nurses from diverse contexts and work environments to gain deeper insights into whether ACT is broadly applicable in nursing settings. It is also important to mention that in our study, no-content-related adaptions were made regarding language and trainiers. Specifically, the original English ACT interventions were translated into the native languages of the nurses (Chinese and German), and the training was delivered by a trainer from the same linguistic and regional background as the nursing participants.

Although the effectiveness of ACT interventions has been demonstrated in both clinical and non-clinical populations, and promising results suggest that ACT is effective in reducing work-related stress across various professional groups [59], previous studies have not focused on comparing the effectiveness of ACT interventions within the same profession — specifically, nurses — across different work contexts and healthcare systems. Our study is the first that explored and descriptively compared outcome patterns of ACT interventions among nurses with different healtcare contexts.

However, the results should be interpreted with caution due to the significant limitations of the study.

A key limitation of this study is the small sample size, which substantially limited statistical power and increased the risk that true between-group effects related to our first hypothesis were not detected. In addition, the small and convenience-based sample restricts the generalizability of the findings. Moreover, the small sample size increases the risk of both Type I and Type II errors. In addition, multiple variables were examined without correction for multiple testing, which may further increase the risk of Type I error. Accordingly, all quantitative findings should be interpreted as exploratory and hypothesis-generating rather than confirmatory. To reduce potential age-related confounding between country samples, participants were matched by age, which further reduced the effective sample size. Given the exploratory nature of the study, this pragmatic approach should be interpreted with caution. This is considered an additional limitation; therefore, non-parametric or Bayesian methods may be considered in future studies with larger samples. Although participants resembled typical nursing professionals in Switzerland and Hong Kong, the results should be interpreted as preliminary and confirmed in larger, more representative samples. Another limitation is the absence of a control group, which precludes causal conclusions. However, as our study was exploratory in nature, the focus was primarily on gaining initial insights into the feasibility and effectiveness of the intervention rather than establishing causal relationships. Further research is needed to replicate the findings and determine causal links between the intervention and improvements in mental health. A further limitation is that we did not systematically track how often participants practiced the exercises between workshops. Consequently, we cannot determine whether this variable contributed to additional variance in the outcomes. Future studies should therefore assess the frequency of at-home practice to evaluate whether this factor plays a crucial role in improving the examined outcomes.

## Conclusion

This study contributes to the growing evidence supporting the potential usefulness of ACT interventions for nursing professionals and provides initial indications that ACT interventions may be applicable among nurses from different healtcare contexts.

The findings suggest that these interventions can lead to improvements in emotional exhaustion, as well as reductions in stress, anxiety, and depressive symptoms. Additionally, the study provides further evidence that ACT interventions can enhance psychological flexibility, which is considered a key process variable in ACT. Given the limited available research on the effectiveness of ACT among nurses with different cultural backgrounds, our findings should be replicated among nurses that differ in culture, to establish more definitive conclusions.

## Supplementary Information

Below is the link to the electronic supplementary material.

Supplementary Material 1: Additional file 1: .pdf; Interview protocol; semi-structured interview guide

Supplementary Material 2: Additonal file 2: .pdf; Evaluation questionnaire; evaluation questionnaire for the proposed interventions in the focus groups

Supplementary Material 3: Additional file 3:. .pdf; Table of content for the workshops; content and schedule of the conducted ACT workshops

Supplementary Material 4: Additional file 4: .pdf; Non_significant_results_figures; Figures presenting non-significant results

## Data Availability

The data supporting this study’s findings are available from the corresponding author upon reasonable request.

## Abbreviations

ACT: Acceptance and Commitment Therapy
HCW: Health care workers
COVID-19: CoronaVirus Disease 2019
EE: Emotional exhaustion
DP: Depersonalization
PA: Personal accomplishment
PsyFlex: Psychological Flexibility
ANOVA: Analysis of Variance
