# Supplementary material for: Improving nurses’ mental health through an online Acceptance and Commitment Therapy intervention: an exploratory pilot study across two healthcare contexts
Source: BMC Nurs. 2026 May 13;25:608. doi: 10.1186/s12912-026-04587-y (PMC13352774; doi:10.1186/s12912-026-04587-y)
Supplement: Supplementary file 1 — Supplementary Material 1: Additional file 1: .pdf; Interview protocol; semi-structured interview guide [file 12912_2026_4587_MOESM1_ESM.pdf]

Welcoming Text and Study information

Dear participant,

Thank you for participating in this interview. The study is conducted by the Department of Clinical Psychology and Intervention Science under the direction of Prof. Dr. Andrew Gloster at the University of Basel in collaboration with Prof. Chong of the Nethersole School of Nursing, The Chinese University of Hong Kong.

The aim of the interview is to determine how nurses can be helped to perform their jobs with less harmful stress and more well-being. In order to design an intervention that is most beneficial to you, we are gathering information from you at the beginning. If you are willing, we will show you the results of whatever we derive and then adjust it if you have further suggestions or requests.

The first step in was to collect data from a survey. Now we wish to follow that up with a more detailed information based on your experiences. With you, we hope to identify work related stress factors and needs of nurses during COVID-19 and in general. Furthermore, we want to investigate the psychological reactions of nurses with perceived barriers, coping strategies and motivators.

In the present study, you will take part in an interview with open questions. The total duration of the course is maximal one hour. The participation in the study is not associated with any health risks. The interview will ask questions of personal nature, such as questions concerning your well-being and coping strategies.

All collected data will be analyzed anonymously and used exclusively by selected persons of the study team for scientific purposes. The results of the study are summarized anonymously in a group and published scientifically without any reference to a specific person.

Your participation in this study is voluntary. Any time during the interview, You can withdraw at any time and no disadvantage. After the completion of the study a subsequent revocation of your data is no longer possible due to the anonymized storage of your data.

This study is funded by the Schweizer Staatssekretariat für Bildung, Forschung und Innovation (SBFI) and the ETH Zürich. This study is being implemented in parallel in Hong Kong and the aggregated results will be compared with those in Hong Kong.

We would now like to start with the first question. Please answer spontaneous and naturally, there are no right or wrong answers.

Vorgehen: 2 Runden

1. **Runde: 8 offene Fragen, clustered in die 5 folgenden Bereiche**

    a. Motivation für Berufswahl
    b. Belastungsfaktoren und Bewältigungsstrategien
    c. Wahrgenommene Unterstützung
    d. Gewünschte Veränderungen
    e. Werte und Commitment

2. **Runde: Follow up probing questions according to the clusters above**

Verbal Instructions 1st Round:

BEFORE Recording:
Welcome to this interview and thank you for your invested time. This interview will take about one hour and consists of two parts. I would like to encourage you to answer as naturally and spontaneously as you can. There are no right or wrong answers. We are interested in your personal experience and your opinion on the questions we ask.

As you have already read in the Study Information, I would like to repeat once more that the interview will be recorded (audio only). After transcribing the audio files, they will be deleted immediately and the anonymity of your data is guaranteed at any time. Do you agree with this? ***(wait for active «Yes»).*** *Then let us start with the recording and the interview.*

Start Recording:

Please re-confirm that you agree to having this interview audio recorded. (wait for active «Yes»).

<u>Interview questions</u>

1. Please tell me about how you have decided to become a nurse. If you were to name ONE motivation, that made you want to become a nurse, what would it be?

2. What are your greatest challenges at work at the moment? How you deal with them?

3. Since the COVID-19 broke out, did you have the impression that you were informed well enough by your supervisor(s) or the hospital management? If not, how did you feel about it?

4. Do you feel supported by your team, your supervisors and/or hospital management? If not, what would need to change so that you feel more supported?

5. If you had the chance to change ONE thing that would help you to deal with the aforementioned problem(s) related to your work, what would it be?

6. If you could receive support from a psychological training, what should this consist of, in your opinion? Or is there any other form of support that would help you? If yes, which one?

7. If you could choose any profession today, would you choose the nurse profession again?

8. Below are areas of life that are valued by some people. I would like to ask you to rate how important each of these areas is for you personally.
On the scale that you can see, 1 means that area is not at all important for you personally. 10 means that area is very important for you. Not everyone will value all of these areas, or value all areas the same. Rate each area according to your own personal sense of importance.

	Not at all important									Very important
Family (other than marriage or parenting)	1	2	3	4	5	6	7	8	9	10
Marriage/couples/ intimate relationship	1	2	3	4	5	6	7	8	9	10
Parenting	1	2	3	4	5	6	7	8	9	10
Friends/social life	1	2	3	4	5	6	7	8	9	10
Work	1	2	3	4	5	6	7	8	9	10
Education/training	1	2	3	4	5	6	7	8	9	10
Recreation/fun	1	2	3	4	5	6	7	8	9	10
Spirituality	1	2	3	4	5	6	7	8	9	10
Citizenship/Community life	1	2	3	4	5	6	7	8	9	10
Physical self-care (diet, exercise, sleep)	1	2	3	4	5	6	7	8	9	10

In the second table I would like to ask you to rate how consistent your actions have been according to your values in the past **7 days**. Please rate each area on the following scale. 1 means that your actions in that area have been completely inconsistent with your values in that area. 10 means that your actions have been completely consistent with your values. Please note that it is completely natural if we don't succeed in acting according to our values at times.

	Not at all consistent according to my values									Totally consistent according to my values
Family (other than marriage or parenting)	1	2	3	4	5	6	7	8	9	10
Marriage/couples/ intimate relationship	1	2	3	4	5	6	7	8	9	10
Parenting	1	2	3	4	5	6	7	8	9	10
Friends/ social life	1	2	3	4	5	6	7	8	9	10
Work	1	2	3	4	5	6	7	8	9	10
Education/training	1	2	3	4	5	6	7	8	9	10
Recreation/fun	1	2	3	4	5	6	7	8	9	10
Spirituality	1	2	3	4	5	6	7	8	9	10
Citizenship/ Community life	1	2	3	4	5	6	7	8	9	10
Physical self-care (diet, exercise, sleep)	1	2	3	4	5	6	7	8	9	10

Verbal Instructions 2nd Round

Now in a second step, I would like to go into a bit of detail for some of the questions I have asked before.

<u>Follow up: Probing questions</u>

Cluster «Challenges and Stress Reactions» (Question 2):

F1:
I would like to talk again about the challenges you experience in your daily routine as a nurse.

What do you think – are those rather ***emotional challenges*** *(e.g., experiencing and dealing with difficult situations like suffering, grief, death or fear with regard to COVID-19, such as getting infected)? Or are those rather* ***organizational challenges*** *(e.g., irregular working hours, not enough staff, etc.) that you experience in your daily routine as a nurse.*

answer: ____________________________________

F2:
Could you say how many percent, approximately, those challenges are of emotional nature for you and how many percent they are organizational?

answer: ____________________________________

F3:
I will now give you some examples for emotional challenges that you might be experiencing in your professional routine as a nurse. Some of them will be related to the current COVID-19 situation in particular.
Please rate how the following statements apply to you personally, using the scale from "not at all" to "extremely" applicable.

	Not at all applicable	Rather not applicable	Somewhat applicable	Applicable	completely applicable
Dealing with suffering, illness and death	1	2	3	4	5
Confrontation with ethical decisions (e.g., triage in treatment of patients, life-preserving measures)	1	2	3	4	5
A feeling of stress because the current	1	2	3	4	5

situation is unpredictable					
Fear of own infection/ infecting someone in your social environment	1	2	3	4	5

F4:
Since COVID-19, has the way you experience stress changed? In what way?

F5:
The challenges you are confronted with can lead to stress reactions. Do you know such reactions? Which ones do you experience?

*answer:*______________________________

F6:
I will now additionally name some typical reactions that people may experience related to stress. Please rate to what degree each reaction applies to you. For that I will show you the following scale.

	never	rarely	sometimes	often	very often
Headaches	1	2	3	4	5
Sleeping problems	1	2	3	4	5
Worries/Anxiety/Uncertainties	1	2	3	4	5
Changes in your eating habits (eating more or less)	1	2	3	4	5
Impact on my social network (e.g., fewer motivation to meet up because I am too tired)	1	2	3	4	5
Others (_________________)	1	2	3	4	5
Others (_________________)	1	2	3	4	5

Cluster «Desired Changes» (Frage 4, 5, 6):

F7:
In the first part of this interview, I have asked you what changes you would wish for so you could better deal with the challenges you experience in your professional routine as a nurse.
In the following I will give you some options as suggestions for change measures. Please indicate how supportive or helpful you think each change measure would be for you to better deal with the challenges you experience as a nurse.
For that I will again show you a scale on which you can rate from «not at all helpful» to «extremely helpful».

	Not at all helpful	Not helpful	Somewhat helpful	Helpful	Very helpful
More psychological care services (e.g., possibility to speak to a psychologist during working hours)	1	2	3	4	5
More team meetings/discussions where everyone can share their difficulties and discuss them	1	2	3	4	5
More feedback by and communication with my superior	1	2	3	4	5
More appreciation of my work	1	2	3	4	5

F8:
As you know, in this study we are aiming to develop an online-based support tool which should help nurses deal with difficulties in their professional routine.
I will now name some possible aspects that could be part of what this support tool can offer you. Please rate how helpful you think those offers would be in better dealing with difficulties in your professional routine.

	Not at all helpful	Not helpful	Somewhat helpful	Helpful	Very helpful
Mindfulness exercises	1	2	3	4	5

Motivation training	1	2	3	4	5
Relaxation training	1	2	3	4	5
Learning to relate differently to difficult thoughts and feelings	1	2	3	4	5
Learning skills to help you act in ways according to your values, which is currently difficult for you	1	2	3	4	5

End:

F9:
Now to end this interview, we would like to give you a voice – a voice as a nurse. If you could give a statement to the public at the moment, what would it be? Would you like your statement to be published anonymously on our website?

F10:
Now, is there anything more you would like to say to end this interview?

Verbal Instructions End:

We are now done with the interview.
Eveline Frey will contact you to provide your compensation.

In a next step, you will be contacted again this spring. We will then provide you with a manual for the online-based support program which you can test and evaluate.

If you have any questions you can contact Eveline Frey any time.

Thank you so much for your participation.
