# Supplementary material for: Improving nurses’ mental health through an online Acceptance and Commitment Therapy intervention: an exploratory pilot study across two healthcare contexts
Source: BMC Nurs. 2026 May 13;25:608. doi: 10.1186/s12912-026-04587-y (PMC13352774; doi:10.1186/s12912-026-04587-y)
Supplement: Supplementary file 2 — Supplementary Material 2: Additonal file 2: .pdf; Evaluation questionnaire; evaluation questionnaire for the proposed interventions in the focus groups [file 12912_2026_4587_MOESM2_ESM.pdf]

Akzeptanz- und Commitment (ACT) Trainingsprogramm für Pflegefachpersonen

Delphi-Evaluationsfragebogen

Part A — Persönliche Angaben

1) Geschlecht:	☐ männlich ☐ weiblich
2) Alter:	Jahre
3) Derzeitige Abteilung (bitte genaue Angabe):	z.B. Herz IPS, HNO Bettenabteilung, ect.
4) Arbeitserfahrung in Jahren (inkl. Ausbildung):	Jahre
5) Erfahrung bei der Pflege/Betreuung von COVID-19 infizierten Patienten:	☐ Ja, ich habe für Monate mit COVID-19 Patienten gearbeitet.
	☐ Nein, ich habe bis jetzt keine COVID-19 Patienten betreut/gepflegt.

Part B — Bevorzugte Struktur für das Trainingsprogramm

Stellen sie sich vor, Sie sind ein Teilnehmer des Gruppenprogramms "Akzeptanz- und Commitment Trainingsprogramm für Pflegefachpersonen", in welchem es darum geht, Burnout Symptome zu reduzieren und das Wohlbefinden von Pflegefachpersonen, welche insbesondere COVID-19 Patienten betreuen, zu steigern. Bitte bewerten sie die folgenden Aussagen, welche sich auf den Rahmen und die Struktur des Programms beziehen.

(Hinweis: 1 = stark ablehnend, 7 = stark zustimmend)

	Stark ablehnend	Mässig ablehnend	Etwas ablehnend	Weder noch	Etwas zustimmend	Mässig zustimmend	Stark zustimmend
1) Es ist besser, halbtägige Workshops als wöchentliche Sitzungen durchzuführen (z. B. 2x2-3h oder jede Woche 1h).	1 ☐	2 ☐	3 ☐	4 ☐	5 ☐	6 ☐	7 ☐
2) Das Programm soll vorzugsweise an Wochenenden im Vergleich zu Wochentagen durchgeführt werden.	1 ☐	2 ☐	3 ☐	4 ☐	5 ☐	6 ☐	7 ☐
3) Persönliche Interaktionen zwischen der Leiterin/dem Leiter des Trainingsprogramms und den Teilnehmern sind während der ACT-Workshops unerlässlich.	1 ☐	2 ☐	3 ☐	4 ☐	5 ☐	6 ☐	7 ☐
4) Das Tempo der Sitzungen sollte genug langsam sein, damit Zeit für die Diskussion/Besprechung der Teilnehmer nach Abschluss jeder ACT-Übung vorgesehen werden.	1 ☐	2 ☐	3 ☐	4 ☐	5 ☐	6 ☐	7 ☐
5) Es ist wichtig, immer Zugang zu den ACT Übungsmaterialien zu haben.	1 ☐	2 ☐	3 ☐	4 ☐	5 ☐	6 ☐	7 ☐

Haben Sie weitere Vorschläge oder Kommentare zur Struktur des Trainingsprogramms?

«Bitte notieren Sie Ihre Vorschläge und Kommentare hier...»

Part C — Bevorzugter Inhalt des Trainingsprogramms

1. Achtsamkeit

Wozu?

In den Interviews wurde häufig von der Schwierigkeit berichtet, nach der Arbeit nicht „abschalten" zu können. Wir vermuten, dass *Achtsamkeitsübungen* den Pflegefachpersonen dabei helfen können, nach der Arbeit bewusst im gegenwärtigen Moment anzukommen und so präsenter zu leben.

Beispiele für Achtsamkeitsübungen:

a. Verschiedene geleitete Achtsamkeitsübungen, in denen die Aufmerksamkeit auf einzelne Teile des Körpers („Body-Scan") sowie die Atmung gerichtet wird. Teilnehmende werden dazu aufgefordert, ihre Gedanken einfach kommen und gehen zu lassen, ohne diese zu bewerten.
b. Achtsames Essen von z.B. einer Rosine, dabei werden auf verschiedene sensorische Informationen geachtet (Aussehen, Haptik, Geruch, Geschmack und weitere). Ziel: Erkenntnis, dass man sich die meiste Zeit in einem „Autopilot-Modus" befindet, anstatt bewusst im „Hier und Jetzt" zu sein.
c. Achtsamkeitsübungen als Hausaufgabe zwischen den zwei Workshops.

(Hinweis: 1 = stark ablehnend, 7 = stark zustimmend)

	Stark ablehnend	Mässig ablehnend	Etwas ablehnend	Weder noch	Etwas zustimmend	Mässig zustimmend	Stark zustimmend
Die oben genannten Achtsamkeitsübungen sind für mich hilfreich/ nützlich, um im Moment bewusster zu leben.	1 ☐	2 ☐	3 ☐	4 ☐	5 ☐	6 ☐	7 ☐

Haben Sie weitere Vorschläge oder Kommentare zum Inhalt dieser Übung?

«Bitte notieren Sie Ihre Vorschläge und Kommentare hier...»

2. Werte & Commitment

Wozu?

In den Interviews wurde häufig berichtet, dass es im Alltag schwierig ist, die „Dinge“ zu tun, die einem wirklich wichtig sind. Beispielsweise: Zeit für soziale Kontakte zu haben, den persönlichen Interessen/Freizeitaktivitäten nachgehen, sich um die eigene Gesundheit kümmern (Erholung, Sport, etc.). Also eine intensive Beschäftigung mit den eigenen Werten, das heisst, sich vertieft damit auseinandersetzten, was einem im Leben wirklich wichtig ist und Ziele zu formulieren, wie man im Alltag konkret nach diesen Werten leben kann (wertorientierte Lebensweise). Wenn es gelingt, sich im Alltag, vermehrt den Dingen zuzuwenden, die einem wirklich wichtig sind, trägt dies zu einem erfüllteren Leben bei und wirkt somit wie ein „Gegengewicht“ zu den Belastungen, mit welchen wir im Alltag konfrontiert sind.

Beispiele für Übungen zum Thema Wertearbeit:

a. **Eine gedankliche Reise in die Zukunft (z.B. zum eigenen 80. Geburtstag) machen:** Was würde man sich wünschen, was die eigenen Freunde und Familienmitglieder am 80. Geburtstag über einem selbst sagen würden? Was wäre mir wichtig, wenn ich mit 80 Jahren auf mein Leben zurückblicke?
b. **Werte-Karten Übung:** Werte für einen Lebensbereich (z.B. Partnerschaft, Freizeit, Freunde, ect) bestimmen, die einem besonders wichtig sind. Dazu werden jedem Teilnehmer 54 Karten mit verschiedenen Werten (z.B. Mutig sein, bewundert werden, mit Anderen verbunden sein, für Andere sorgen, Spass und Vergnügen im Alltag erleben, sportlich aktiv sein) zur Verfügung gestellt. Anhand dieser Wertekarten, formuliert man anschliessend konkrete Ziele, um eine wertorientierte Lebensweise im Alltag zu erlangen.

(Hinweis: 1 = stark ablehnend, 7 = stark zustimmend)

	Stark ablehnend	Mässig ablehnend	Etwas ablehnend	Weder noch	Etwas zustimmend	Mässig zustimmend	Stark zustimmend
Wertearbeit und präzise formulierte Ziele sind für mich hilfreich/ nützlich, um mehr nach den eigenen Werten zu leben und zu handeln.	1 ☐	2 ☐	3 ☐	4 ☐	5 ☐	6 ☐	7 ☐

Haben Sie weitere Vorschläge oder Kommentare zum Inhalt dieser Übung?

«Bitte notieren Sie Ihre Vorschläge und Kommentare hier...»

---

3. Akzeptanz und kognitive Defusion:

Wozu?

In den Interviews wurde häufig berichtet, dass durch die vielen Belastungen bei der Arbeit oft schwierige Gefühle und störende Gedanken (z.B. nicht abschalten können oder immer an schwierige Situationen am Arbeitsplatz denken zu müssen) im Alltag auftauchen. Insbesondere die Gefühle Stress, Wut, Ärger, Unzufriedenheit und Ängste, wurden von Interviewteilnehmern oft genannt. Viele gaben an, dass es für sie schwierig ist, mit diesen Gefühlen und Gedanken umzugehen und deshalb manchmal „Dinge" tun, die sie nicht tun würden, wenn diese Gefühle und Gedanken nicht oder weniger vorhanden wären (z.B. gereizt zu reagieren auf Partner, den Kindern oder Freunden; schneller weinen, weil man nichts ertragen kann oder vermehrt Alkohol zu trinken, weil man diese Gefühle loswerden oder gedanklich „abschalten" möchte.

Ein zentrales Element bei der Akzeptanz- und Commitment Therapie ist es, einen verbesserten Umgang mit belastenden Gedanken und schwierigen Gefühlen zu erlernen. Es gibt Strategien, wie man sich besser von solch belastenden Gedanken distanzieren kann («kognitive Defusion»). Zudem trägt die Akzeptanz von schwierigen Gefühlen dazu bei, dass sich diese weniger belastend auf unser Erleben auswirken und unser Verhalten und Handeln im Alltag weniger von diesen Gefühlen beeinflusst werden.

Beispiele für Defusions- und Akzeptanz-Übungen:

a. **Vermittlung der "Passagiere-im-Bus-Metapher":** Mittels verschiedenen Methaphern, vermittelt die Leiterin des Workshops, wie wir gedanklich Distanz zu störenden und belastenden Gedanken schaffen können. Fokus bei der Bus-Methapher: Du bist der oder die Busfahrer/in und bestimmst die Richtung, anstatt dich von den störenden Passagieren (->störenden Gedanken) lenken zu lassen. Wir können die Passagiere (->Gedanken) nicht einfach aus dem Bus (-> unserem Kopf) werfen und gewaltsam loswerden. Stattdessen können wir lernen, sie zu akzeptieren und uns bewusst machen, dass es schlussendlich „nur" Passagiere (->Gedanken) sind, welche uns zwar begleiten, aber nicht lenken können.
b. **„Watching-your-thoughts" Übung:** Hinderliche Gedanken/Gefühle identifizieren, aufschreiben und anhand verschiedener kleiner Übungen herausarbeiten, dass eine gesunde Distanz zu diesen Gedanken und Gefühlen wichtig ist. So können diese keinen ungünstigen Einfluss auf das eigene Verhalten mehr ausüben.

(Hinweis: 1 = stark ablehnend, 7 = stark zustimmend)

	Stark ablehnend	Mässig ablehnend	Etwas ablehnend	Weder noch	Etwas zustimmend	Mässig zustimmend	Stark zustimmend
Verschiedene Übungen, die helfen, schwierige Gedanken und Gefühle anzunehmen und loszulassen, sind für mich hilfreich/ nützlich, um mehr Kontrolle über die eigenen Gefühle und das eigene Verhalten zu erlangen.	1 ☐	2 ☐	3 ☐	4 ☐	5 ☐	6 ☐	7 ☐

Haben Sie weitere Vorschläge oder Kommentare zum Inhalt dieser Übung?

«Bitte notieren Sie Ihre Vorschläge und Kommentare hier...»

4. Werte im Team und im Arbeitsalltag in der Pflege

Wozu?

In den Interviews wurde sehr deutlich, wie wichtig das eigene Team im Arbeitsalltag von Pflegefachpersonen ist. Unter anderem wurde berichtet, dass das Team eine sogenannte „Pufferfunktion" hat und so dem Einzelnen hilft, besser mit dem Stress während des Arbeitsalltags, umgehen zu können. Da Werte und eine wertorientiertere Lebensweise nicht nur das eigene Leben positiv beeinflussen, sondern sich auch positiv auf Interaktionen im Team und das Teamklima auswirken, gehen wir davon aus, dass die vertiefte Auseinandersetzung mit Werten im Team und einer wertorientierten Zusammenarbeit im Team ebenfalls hilfreich beim Umgang mit arbeitsplatzbezogenem Stress sein wird.

Beispiele für Wertearbeit bezogen auf Team:

a. **Gruppendiskussion**, verschiedene Fragen zu Werten und wertorientierter Zusammenarbeit diskutieren und gemeinsam erarbeiten:
   - Was ist uns als Team wichtig?
   - Wie wollen wir als Team interagieren?
   - Wie gelingt es uns, dass wir uns im Arbeitsalltag vermehrt so verhalten können?

(Hinweis: 1 = stark ablehnend, 7 = stark zustimmend)

	Stark ablehnend	Mässig ablehnend	Etwas ablehnend	Weder noch	Etwas zustimmend	Mässig zustimmend	Stark zustimmend
Eine Gruppendiskussion über das werteorientierte Leben im Team ist hilfreich/ sinnvoll, um Wege zur Verbesserung des Teamklimas zu erarbeiten.	1 ☐	2 ☐	3 ☐	4 ☐	5 ☐	6 ☐	7 ☐

Haben Sie weitere Vorschläge oder Kommentare zum Inhalt dieser Übung?

«Bitte notieren Sie Ihre Vorschläge und Kommentare hier...»

5. Weitere mögliche Inhalte:

Gibt es weitere Komponenten, die Ihrer Meinung nach wichtig sind, um das Burnout des Pflegepersonals zu reduzieren, die aber in diesem Trainingsprogramm nicht enthalten sind oder erfüllt werden?

«Wenn ja, bitte notieren Sie Ihre Vorschläge und Kommentare hier...»

Ende der Evaluation – vielen Dank für Ihre Teilnahme.
