# Supplementary material for: Improving nurses’ mental health through an online Acceptance and Commitment Therapy intervention: an exploratory pilot study across two healthcare contexts
Source: BMC Nurs. 2026 May 13;25:608. doi: 10.1186/s12912-026-04587-y (PMC13352774; doi:10.1186/s12912-026-04587-y)
Supplement: Supplementary file 3 — Supplementary Material 3: Additional file 3:. .pdf; Table of content for the workshops; content and schedule of the conducted ACT workshops [file 12912_2026_4587_MOESM3_ESM.pdf]

ACT-Workshop für Pflegefachpersonal Kantonsspital Graubünden 10. Und 17. Juni 2021

Workshop Day 1: June 10 2021, 3 pm – 6.30 pm

Intervention	Task	Content	Reference Handout	Material	Duration	Notes for Trainer
	Begrüßung	- Introduction of trainer and participants,<br>- Structure workshop (emphasize safe space),<br>- short introduction to content + goal			15 min	
Intro: Acceptance/ Mindfulness	„Physicalizing Exercise“	- Putting yourself in stressful situation;<br>- visualization of stress<br>- Perceive how mood changes when we accept it; perceive that mood does not stay the same	S. 134		10min	
Acceptance/ Mindfulness	Group discussion	- short discussion about experiences/ impressions during exercise<br>- How did the participants feel?<br>- Were they able to switch off their thoughts?<br>- Do they perhaps even have experience with mindfulness - if so, which?			5-10 min	
Introduction	Two-Skills Diagramm	- Present diagram and its components			10min	
Mindfulness	-Body-Scan or<br>-«Mindful-eating» rasin exercise<br>(let participants chose)	- Paying attention to different parts of the body + breathing<br>- Perceive thoughts, do not evaluate them and let them pass by again<br>- pay attention to different sensory info (appearance, feel, smell, etc. of the raisin).	(Instruktionen S. 77)	Rasin, aple, ect)	5 min	
Mindfulness	Group discussion	- Im Plenum diskutieren<br>- Reflect on experiences and then compile them in the plenum			10min	- 5min Break-out groups<br>- 5min plenums-discussion
		*** PAUSE (10min) ***				

Values	Thought exercise 80th birthday<br><br><br>Werte theoretischer Input Values theoretical input<br><br><br><br>Buffer: Compass methaphor	- Mental journey to your own 80th birthday,<br>- What do you wish friends would say about you?<br>- How would you like to be remembered?<br><br>- Transition to values + value-oriented action<br>- What are values (vs. goals)?<br>- lived values = desired direction in life, personal strengths,...während: Ziel = spezifisches Ergebnis<br><br>- Compass metaphor (values are not far away in the distance, but show us the direction, so that we can be guided by them in every moment)	Harris, Buch S. 324	Build Break-out sessions (groups of two)	15 min	
Commitment	Advantages of value-driven action	- - What advantage does it bring to be guided by your values?			5min	
Values	Values card sort task (top 5)	- Worksheets with 5 different areas of life (including Relationship, Personal Development...).<br>- Select 1 of these areas of life + define values with provided value cards<br>- Break down values further and further into key words		values cards will be sent to participants in advance by mail	15min	
Values	Group discussion about impressions	- Eindrücke in Gruppe teilen<br>- (nicht explizit die eigenen Werte, sondern Erfahrungen die man bei der Wahl gemacht hat)			5-10min	
**********PAUSE *******<br>(10 min)**						
Commitment	3 Translating values into actions	- Basis for homework at the end of the workshop<br>- Brainstorming regarding different value-oriented actions that could be implemented within the next week(s).<br>- "Bring this value to life!"<br>- collect as many simple, small actions as possible (size of the action itself is not important, the main thing is that it is in line with the value)		Worksheet/ Handout	15min	

		- Choose 3 actions that you want to do within the next week + write them down in the worksheet<br>- Formulate actions as specifically as possible				
Values	Work out inner obstacles	- Intro: Exercise: book metaphor: valued-based action are not possible, as long as we "hold the book" as we are fused with painful thoughts and feelings<br>- Anticipate barriers that might prevent values-oriented action.<br>- Group discussion: what might prevent one from representing + acting on one's values?<br>- Classification into inner + outer barriers<br>- (external ones only difficult to influence focus on overcoming the internal barriers with one's own abilities)	(S.93)	Whiteboard to sum up ideas	15min	
Values	Inner obstacles	- Work out own obstacles on worksheet		Worksheet, handout	5min	
Conclusion WS one	«Two-sheet technique»	- write "values" + "hindering thoughts/moods/feelings" on 2 large sheets of paper<br>- Distance to camera, put "values" in foreground = goal of ACT workshop<br>- Goal: to align our own behavior more with our values (the things that are important to us) instead of being controlled by hindering thoughts & feelings (= barriers/ obstacles).<br>- this is achieved by accepting and consciously perceiving our inner barriers (instead of trying to get rid of them = avoidance + costs a lot of energy)	(S.96)	2 A4 sheets, labeled	10min	
Home assignments	Übungsaufgaben erklären	1) 1) Mindfully perform the +- 3 selected value-driven actions over the next week.<br>a. Sticker/ sticky note as a small reminder<br>b. pay close attention to what thoughts + feelings arise when pursuing these actions<br>c. observe how the environment reacts to them / other consequences of these value-based actions<br>2) 2) Perform mindfulness exercises	(S. 98)	Send a link to 2-3 different mindfulness exercises (Body-Scan, mindful breathing)	10min	

		a. +- 3x in the next week (approx. 10min per exercise)<br>b. helps to create a greater awareness of the present moment				
End	Puffer: Fragen, Feedback & Reflektionen	- End with renewed short reference to 2-skills diagram<br>- time buffer<br>- Space for questions and suggestions			5min	

Workshop Day 2: June 17 2021, 3 pm – 6.30 pm

Introduction	Welcoming	- - Express joy about renewed workshop<br>- - short repetition 2 skills diagram			5min	
Values	Discussion of exercise («Value consistency»)	- in small break-out groups to become more personal<br>- (specify questions that can be discussed)	S.111 / S.153	Questions in HO will be discussed	10min	
		⍰ In a plenary session, compile experiences<br>- - e.g. what prevented me from behaving according to my values?			15min	
ACT / Acceptance & cognitive defusion	Passenger in the bus methaphor	- - Participants as bus driver in the "bus of life", full of monster passengers (=feelings, thoughts)<br>- - some well willing, others questioning one + demanding change of direction + power to decide<br>- - WE as bus driver, determine the direction instead of being steered by passengers<br>- - Goal Program: become aware of these passengers with the help of mindfulness and thus be better able to determine if and how we want to be guided by them in our actions.<br>- - "We are at the wheel and we can steer the bus! In the direction that is important to us."		Show picture oft he methaphor	10min	
ACT	Group discussion about dealing with barriers	- Inquire thoughts on this metaphor<br>"Why don't we as bus drivers just kick these passengers off the bus / is it that easy?"			15min	

		- Conclude, "fellow passengers are just thoughts and feelings, trying to get rid of them with all our might is not an option, only makes them stronger; ultimately they are just thoughts and feelings, we don't have to do what they ask of us!"<br>- "Do these difficult passengers also exist in ourselves? What are these passengers, what do they want from us?"<br>- "It takes mindfulness: to become aware of the passengers and know their influence<br>- Defusion: to be able to take a step 'back'<br>- - Acceptance: that these passengers are part of our lives! "				
			********** Break ******* (10 min)**			
Defusion	Defusions- exercise: «Watching your thoughts exercise»	- Ask participants to write one or more hindering thoughts or feelings in large letters on a blank sheet of paper and fill it in.<br>- Ask participants to hold sheet firmly in both hands + hold directly in front of face so that it almost touches nose<br>- Share thoughts in plenary<br>- Ask participants to slowly remove sheet from face Share impressions again (What changes now with some more distance to thoughts and feelings?)<br>- Workshop goal = develop mindful awareness to create healthy distance, so thoughts can be seen for what they are<br>- look at thoughts instead of looking at the world through our thoughts<br>- Ask participants to hold leaf up close in different ways, see what happens<br>- Note: holding long arms outstretched = very strenuous, much easier to discard thoughts (point out that constant focus on obstructive thoughts takes a lot of energy)<br>- Translated with www.DeepL.com/Translator (free version)In conclusion: Putting leaf on lap detaching from thoughts		Empty sheet	15 min	- Give example of hindering thoughts Maybe put microphones directly in front of it so that you can also talk with a sheet in front of your face.

		doesn't mean pushing them away or removing them from oneself, instead: becoming mindfully aware of these thoughts no matter how undesirable/less helpful				
**Buffer:** values or Defusion techniques	Value consistency<br>Values-based actions	- Fill out worksheet "Assessing value consistency".<br>- Behavior that was consistent with value vs. behavior that was not consistent with value<br>- How important is this value still? (1-10 scale)<br>- How much was behavior consistent with value (1-10 scale)?	S.241		15-20min	
********** Break ******* (10 min)**						
Values	Group discoussion	- - What would value-oriented life in the team be like? What would that look like?<br>- - What would be benefits of value-based team behavior?<br>- - What would help value-based behavior as a team?			20min	- 10min in Break-out groups<br>- 10min im Plenum
Values	4-weeks-plan: specify valued based goals and actions	- Write down values in at least 2 areas of life (preferably other areas than in the first workshop)<br>- - Set appropriate short term, medium term and long term goals (4 short term goals that you then want to implement as an exercise in the next 4 weeks)<br>- - + 3 value-guided actions that can be done in the next week in a mindful way<br>- - create small reminders (mobile phone/ stickers/ sticky notes)	(S.141)	Handout	20min	Give tips for implementation (e.g. present example with own value to clarify difference between target + value)
End	conclusion workshop program	- go over the 2-skills diagram again<br>- briefly repeat which strategies have been learned so far			10min	
Buffer	Questions and feedback, further instructions (folow-up survey, calls)				10 min	
