# Supplementary figures and images for: Improving nurses’ mental health through an online Acceptance and Commitment Therapy intervention: an exploratory pilot study across two healthcare contexts
Source: BMC Nurs. 2026 May 13;25:608. doi: 10.1186/s12912-026-04587-y (PMC13352774; doi:10.1186/s12912-026-04587-y)

Plot 6

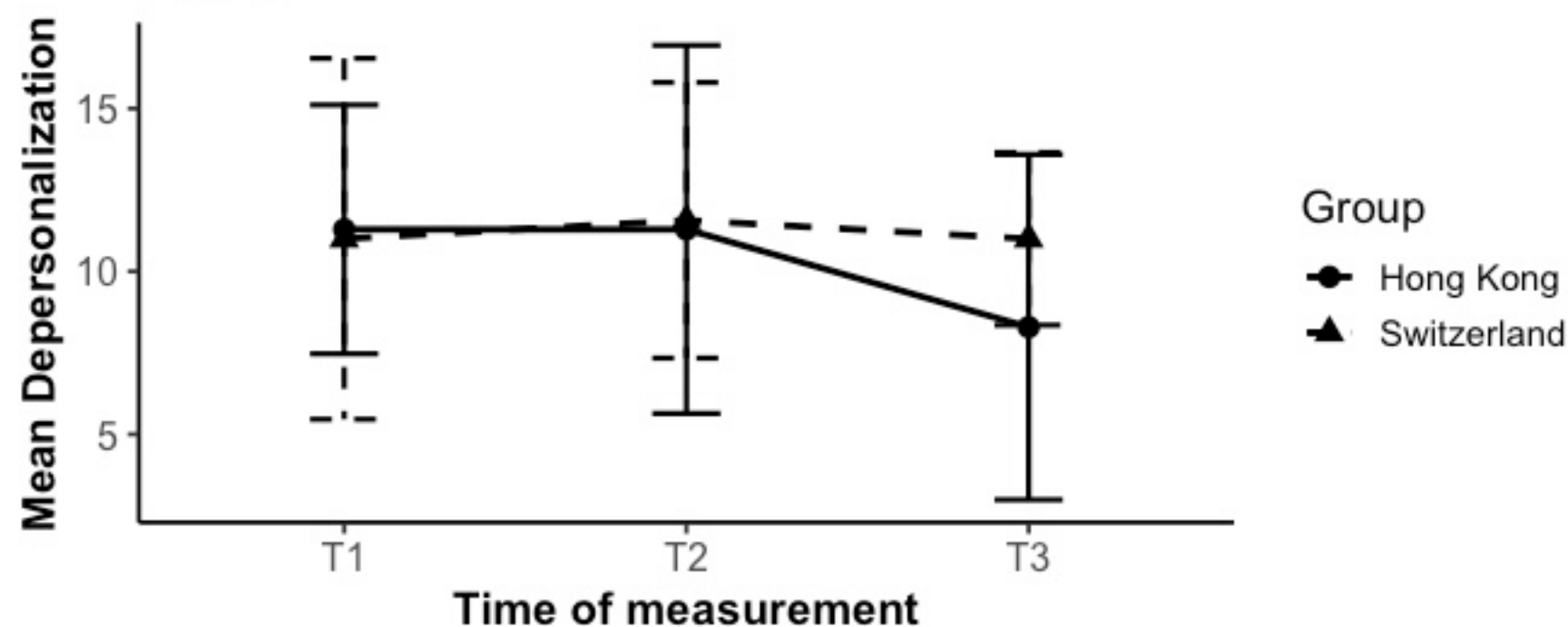

Plot 7

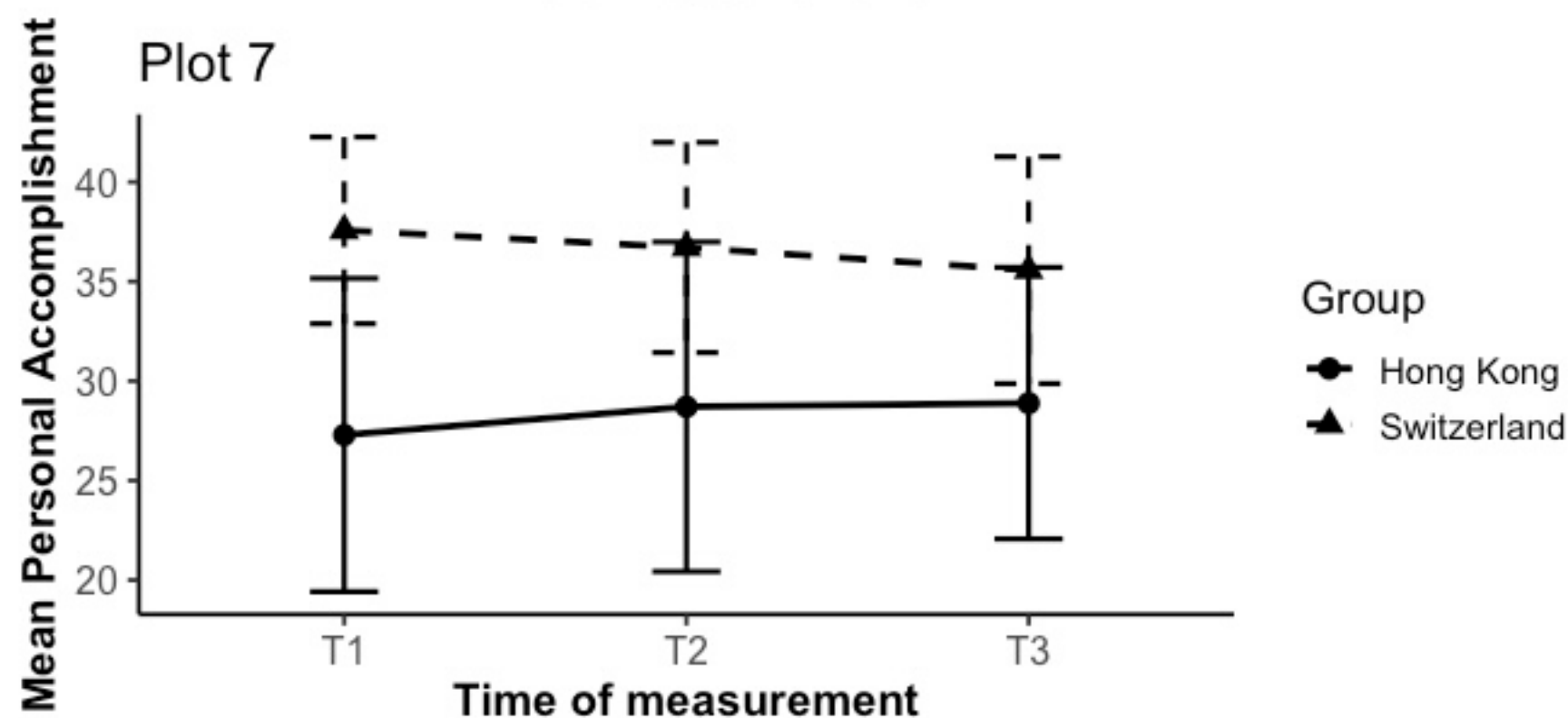

Plot 8

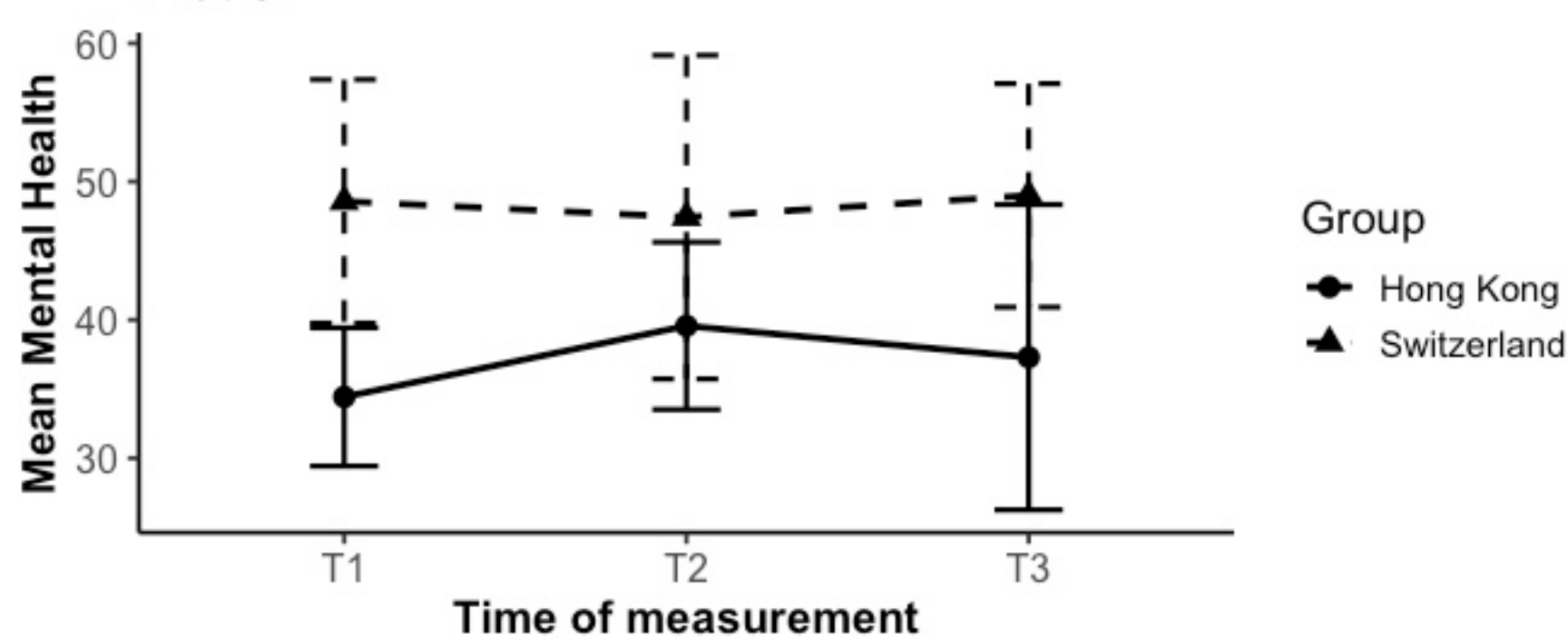

Supplement: Supplementary file 4 — Supplementary Material 4: Additional file 4: .pdf; Non_significant_results_figures; Figures presenting non-significant results [file 12912_2026_4587_MOESM4_ESM.pdf]
